# Case report: Successful treatment of Chidamide in a refractory/recurrent SPTCL with ARID1A mutation on the basis of CHOP plus auto-HSCT

**DOI:** 10.1097/MD.0000000000035413

**Published:** 2023-10-06

**Authors:** Nan Zhang, Shan Zhang, Lei Ma, Ling Qiu, Qing-Li Meng, Jiao Cai, Zhen Xu, Hao Yao, Fang-Yi Fan

**Affiliations:** a Department of Hematology, People’s Liberation Army the General Hospital of Western Theater Command, Chengdu, Sichuan, China; b Department of Pathology, People’s Liberation Army the General Hospital of Western Theater Command, Chengdu, Sichuan, China.

**Keywords:** AT-rich interaction domain 1A (*ARID1A*), autologous hematopoietic stem cell transplantation (auto-HSCT), case report, Chidamide, subcutaneous panniculitis like T-cell lymphoma (SPTCL)

## Abstract

**Rationale:**

Subcutaneous panniculitis like T-cell lymphoma (SPTCL) is a rare primary cutaneous lymphoma that belongs to peripheral T cell lymphomas, of which the overall prognosis is poor. Chidamide, a deacetylase inhibitor, has been approved for the treatment of peripheral T cell lymphomas. However, due to the rare occurrence of SPTCL, it is currently unknown whether Chidamide is effective for all SPTCL patients and whether there are molecular markers that can predict its therapeutic effect on SPTCL.

**Patient concerns and diagnoses:**

The patient was a sixteen-year-old male and underwent subcutaneous nodule biopsy which showed SPTCL. Next-generation sequencing revealed AT-rich interaction domain 1A (*ARID1A*) mutation, and positron emission tomography/computed tomography showed scattered subcutaneous fluorodeoxyglucose metabolic lesions throughout the body.

**Interventions and outcomes:**

During the first 3 CHOP (cyclophosphamide, doxorubicin, vindesine, and prednisone) treatment, the patient relapsed again after remission, and the successive addition of methotrexate and cyclosporine did not make the patient relapsing again. Then, after adding Chidamide to the last 3 CHOP treatment, the patient was relieved again. The patient underwent autologous hematopoietic stem cell transplantation (auto-HSCT) after completing a total of 8 cycles of chemotherapy, and continued maintenance therapy with Chidamide after auto-HSCT. Currently, the patient has been in continuous remission for 35 months.

**Lessons subsections:**

This case is the first report of a refractory/recurrent SPTCL with *ARID1A* mutation treated with Chidamide. The treatment of Chidamide on the basis of CHOP plus auto-HSCT therapy achieved good results, suggesting that *ARID1A* may act as a molecular marker to predict the therapeutic effect of Chidamide on SPTCL patients, which helps to improve the precision of SPTCL treatment and the overall prognosis of SPTCL patients.

## 1. Introduction

Subcutaneous panniculitis like T-cell lymphoma (SPTCL) is a rare subtype of peripheral T cell lymphoma (PTCL), and caused by panniculitis like cytotoxicity α-β T cells infiltrate subcutaneous tissues.^[1]^ The incidence rate of SPTCL is extremely low, accounting for <1% of non-Hodgkin lymphoma.^[2]^ SPTCL usually occurs in young adults, with an average age of 36 years. Patients with SPTCL usually have multiple subcutaneous nodules of 0.5 to 2.0 cm in size located in the lower limbs, upper limbs or trunk, and positron emission tomography/computed tomography (PET/CT) is recommended for disease evaluation.^[3,4]^

At present, there is no standard treatment scheme for SPTCL.^[5]^ Chidamide is a deacetylase inhibitor and has been approved for the treatment of PTCL.^[6]^ However, due to the rare occurrence of SPTCL, there are currently few reports on the treatment of SPTCL with Chidamide. Only 1 case has reported that Chidamide performs well in the treatment of refractory SPTCL.^[7]^ Nevertheless, it is still currently unknown whether all SPTCL patients can be routinely treated with Chidamide and whether there are molecular markers which can predict the efficacy of Chidamide on SPTCL.

In order to search for molecular markers that may predict the therapeutic effect of Chidamide and promote precise treatment and overall prognosis of SPTCL, we report here a refractory/recurrent SPTCL patient with AT-rich interaction domain 1A (*ARID1A*) mutation who achieved continuous remission after the addition of Chidamide on the basis of CHOP (cyclophosphamide, doxorubicin, vindesine, and prednisone) plus autologous hematopoietic stem cell transplantation (auto-HSCT).

## 2. Methods

The study was approved by the Ethics Committee of People’s Liberation Army The General Hospital of Western Theater Command.

The authors confirm that “written informed consent” was obtained from the patient’s legal guardian.

## 3. Patient information

The patient was a sixteen-year-old male who started experiencing intermittent fever 3 months before admission. Ten days before admission, scattered subcutaneous nodules protruding from the skin appeared throughout his body. No previous special medical history, no related diseases in the family.

## 4. Clinical findings

**Figure 1.**
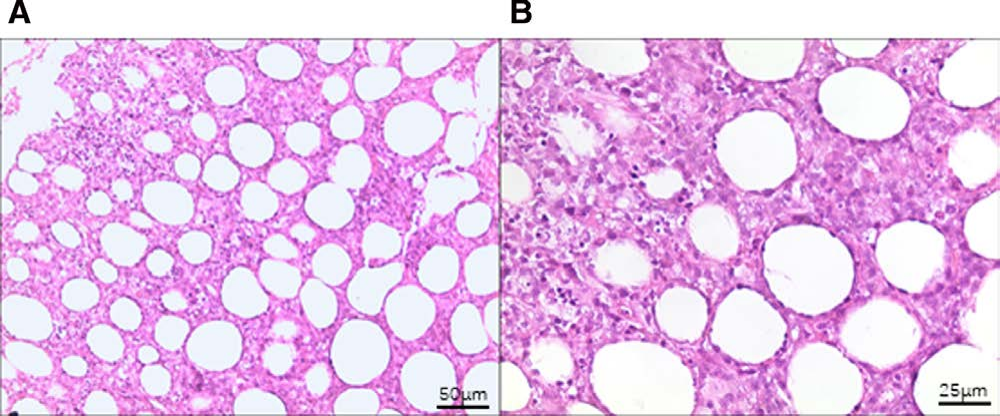
The histopathological features of the SPTCL patient. (A and B) The 200× (A), 400× (B) HE staining pictures of the subcutaneous nodule of the patient’s right costal arch. SPTCL = subcutaneous panniculitis like T-cell lymphoma.

**Figure 2.**
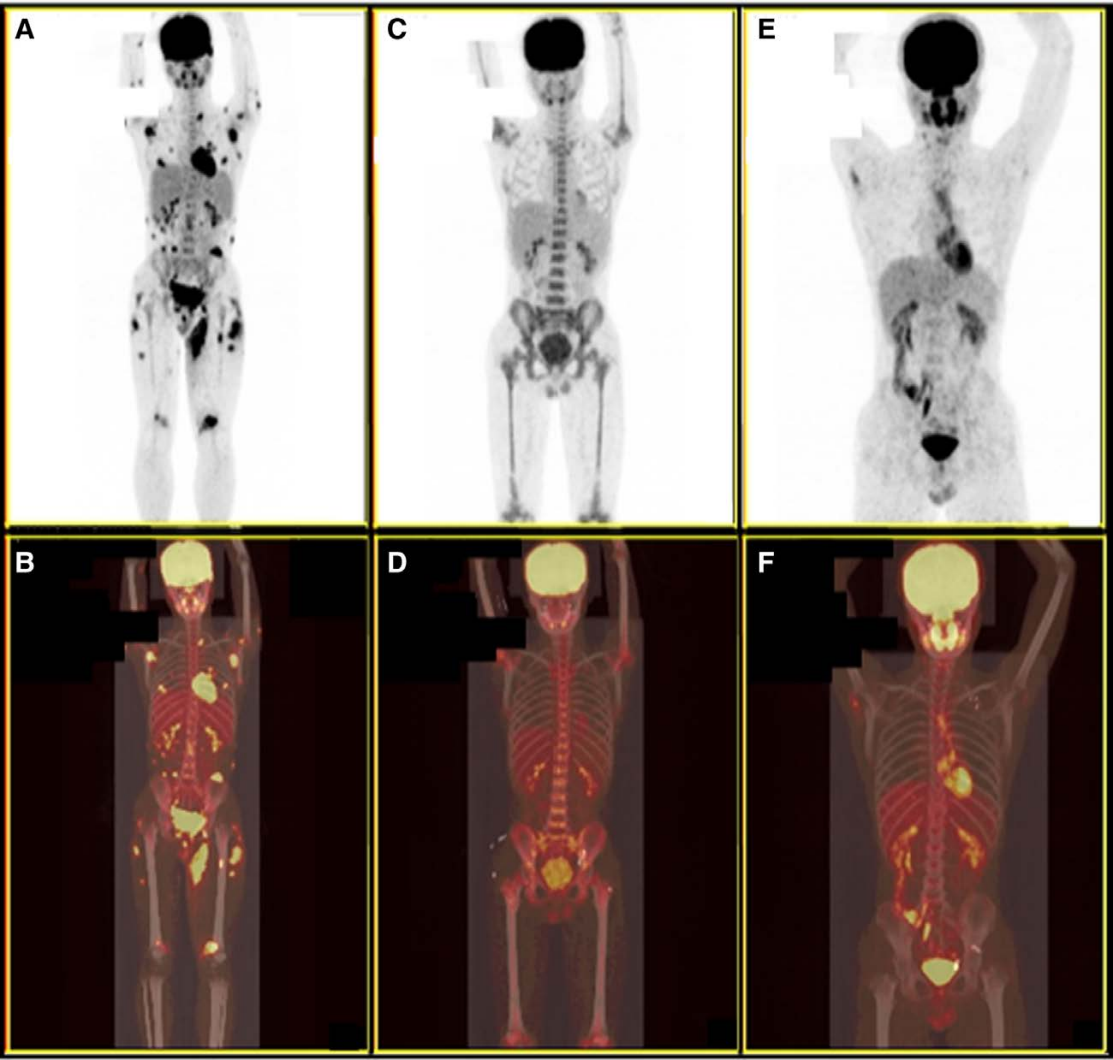
PET/CT images of the SPTCL patient at different time points. (A–F) PET/CT images of this case at the initial diagnosis (A and B), after 8 cycles of chemotherapy (C and D) and 16 months after auto-HSCT (E and F). auto-HSCT = autologous hematopoietic stem cell transplantation, PET/CT = positron emission tomography/computed tomography, SPTCL = subcutaneous panniculitis like T-cell lymphoma.

Auxiliary examinations after admission are described as follows. White blood cell count: 3.48 * 10^9^/L. Red blood cell count: 3.90 * 10^12^/L. Hemoglobin concentration: 105 g/L. Platelet count: 139 * 10^9^/L. Aspartate transaminase: 70.20 IU/L. Lactic dehydrogenase: 996.40 IU/L. Hypersensitive C-reactive protein: 18.77 mg/L. Procalcitonin: 0.10 ng/mL. Ferritin: 1807.80 ng/mL. NK cell activity: 10.39%. Soluble CD25: 2713.80 pg/mL. Bone marrow morphology indicates that bone marrow is not invaded. Biopsy of subcutaneous nodule at the right costal arch showed that there were a large number of lymphocyte-like cells in the subcutaneous fat layer (Fig. 1A and B). Immunohistochemistry: CD8 (+), Ki-67 (+), 70%, CD3 (+), CD5 (+), CD20 (−), Pax-5 (−), GRB (+), TIA (+), CD56 (−). Second generation sequencing: *ARID1A* gene mutation (mutation frequency 45.2%). PET/CT indicates that there are different degrees of increased fluorodeoxyglucose metabolism in multiple subcutaneous fat spaces throughout the body (Fig. 2A and B).

## 5. Diagnostic assessment

This patient was diagnosed as “SPTCL with *ARID1A* mutation.”

## 6. Therapeutic intervention, follow-up and outcomes

**Figure 3.**
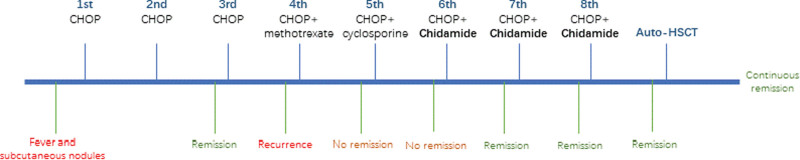
The timeline of this case. One month interval between each chemotherapy and between chemotherapy and auto-HSCT. auto-HSCT = autologous hematopoietic stem cell transplantation, CHOP = cyclophosphamide, doxorubicin, vindesine, and prednisone.

During the first 3 cycles of CHOP, the fever and subcutaneous nodules gradually subsided. However, before the fourth CHOP, the fever and subcutaneous nodules reappeared. After the successive addition of methotrexate and cyclosporine on the basis of CHOP, it has not been alleviated. Therefore, since the sixth CHOP, we added Chidamide, and the patient was relieved again. After completing a total of 8 cycles of chemotherapy, the patient underwent the second PET/CT which indicated that this patient was in complete remission (Fig. 2C and D). Then the auto-HSCT was performed. After pretreatment with etoposide, cytarabine, carmustine, and mephalan scheme, autologous stem cells (total nucleated cell 997.98 * 10^8^, CD34 246.38 * 10^6^) were reinfusion. Platelets and leukocytes were separately transplanted alive at tenth and twelfth day after transplantation. Sixteen months later, the third PET/CT was performed which revealed continuous remission (Fig. 2E and F). The patient is still in regular follow-up. So far, the patient has been in remission for 35 months. The timeline is shown in Figure 3.

## 7. Discussion

For the treatment of SPTCL, studies have shown that most cases can be successfully treated with systemic corticosteroids or immunosuppressants, such as etoposide, cyclosporine, methotrexate.^[8]^ When the disease progresses, conventional doxorubicin-based chemotherapy such as CHOP can be used. Radiotherapy can lead to long-term remission and may play a role in relieving patients with local diseases. HSCT can be carried out in refractory or disseminated cases.^[9]^

There are a few literature reports on potential therapeutic targets and targeted drugs for SPTCL. Maliniemi et al reported that indoleamine 2,3-deoxygenase 1, which induces immune tolerance in the tumor microenvironment, is expressed by malignant cells, CD33+ myeloid-derived suppressor cells and CD163+ tumor-associated macrophages in SPTCL. This report indicating indoleamine 2,3-deoxygenase 1 may act as a new therapeutic target for the treatment of SPTCL.^[10]^ Zhang et al reported that SPTCL patients harboring hepatitis A virus cellular receptor 2 mutations responded well to ruxolitinib targeting inflammatory cytokines, allowing rapid disease resolution and/or long-term maintenance of remission.^[11]^ The mechanism behind this may be related to hepatitis A virus cellular receptor 2 encoding T-cell immunoglobulin mucin 3, which is a critical checkpoint molecule that regulates inflammatory responses and its defection leads to persistent inflammatory responses.^[12]^

Chidamide is one of the histone deacetylase inhibitors, which selectively inhibits histone deacetylase (HDAC) 1, 2, 3, and 10.^[6]^ Chidamide was approved in December 2014 by the China Food and Drug Administration for the treatment of relapsed or refractory PTCL.^[6]^ However, due to the rare occurrence of SPTCL, there are currently few reports on the treatment of SPTCL with Chidamide. Only 1 case has reported that Chidamide performs well in the treatment of refractory SPTCL.^[7]^ This case we report is a refractory/recurrent SPTCL, which achieved continuous remission after the addition of Chidamide on the basis of CHOP plus auto-HSCT, once again confirming the efficacy of Chidamide in SPTCL. However, more particularly, this case was accompanied by a mutation in *ARID1A*, indicating that the good efficacy of Chidamide in this SPTCL case may be related to the *ARID1A* mutation.

ARID1A is an important SWItch/Sucrose Non-Fermentation chromatin remodeling complex subunit, and is the most frequently mutated epigenetic regulator across human cancers.^[13]^ Current studies have reported that ARID1A acts as a tumor suppressor, regulating gene transcription, participating in DNA damage response, and influencing tumor immune microenvironment and signaling pathways.^[14]^ The deletion of *ARID1A* in cancer leads to widespread dysregulation of gene expression in cancer initiation, promotion, and progression.^[15]^ Emerging researches have reported that tumor cells with *ARID1A* mutations can benefit from the treatment of histone deacetylase inhibitors.^[16–21]^ For example, Kuo et al reported that HDAC inhibitors show therapeutic benefit on *ARID1A* depletion-induced pancreas dysfunction.^[19]^ For another example, Park et al reported that human endometrial epithelial cell lines with *ARID1A*-knockout were more sensitive to HDAC inhibitor than their wild type cell controls.^[21]^ This SPTCL case with *ARID1A* mutation we reported here showed continuous remission after treatment with Chidamide, suggesting that SPTCL with *ARID1A* mutation may benefit from treatment with Chidamide. More basic and clinical studies are needed to further clarify the relationship and mechanism between *ARID1A* mutation and the efficacy of Chidamide.

In this SPTCL case with *ARID1A* mutation, the treatment of Chidamide on the basis of CHOP plus auto-HSCT achieved good results, indicating *ARID1A* may serve as a molecular biomarker for predicting the efficacy of Chidamide in the treatment of SPTCL. It is suggested that SPTCL patients should undergo next-generation sequencing. If the mutation of *ARID1A* or other genes involved in regulating epigenetics is found, treatment with Chidamide can be attempted. This case report helps to improve the precision of SPTCL treatment and the overall prognosis of SPTCL patients.

## 8. Patient perspective

The patient agrees to our treatment and has no objections.

## Acknowledgments

We gratefully acknowledge the financial support by the Natural Science Foundation of Sichuan Province [2022NSFSC1330], the Sichuan Science and Technology Programme [2021YJ0145], and the Basic and Frontier Research Project of General Hospital of the Chinese People’s Liberation Army Western Theater [2021-XZYG-C46 to Fang-Yi Fan, 2021-XZYG-B32 to Hao Yao].

## Author contributions

**Conceptualization:** Nan Zhang, Fang-Yi Fan.

**Data curation:** Nan Zhang, Shan Zhang, Lei Ma, Ling Qiu, Qing-Li Meng, Jiao Cai, Zhen Xu, Hao Yao, Fang-Yi Fan.

**Funding acquisition:** Nan Zhang, Hao Yao, Fang-Yi Fan.

**Writing – original draft:** Nan Zhang.

**Writing – review & editing:** Nan Zhang, Fang-Yi Fan.
